# Recent developments in biosensing methods for extracellular vesicle protein characterization

**DOI:** 10.1002/wnan.1839

**Published:** 2022-08-23

**Authors:** Jugal Suthar, Marissa Taub, Randy P. Carney, Gareth R. Williams, Stefan Guldin

**Affiliations:** ^1^ Department of Chemical Engineering University College London London UK; ^2^ UCL School of Pharmacy University College London London UK; ^3^ Department of Biomedical Engineering University of California, Davis Davis California USA

**Keywords:** absorbance, acoustic resonators, biosensing, electrochemical, electrochemical quartz crystal microbalance with dissipation, exosomes, extracellular vesicles, fluorescence, interferometry, plasmon resonance, surface enhanced Raman spectroscopy

## Abstract

Research into extracellular vesicles (EVs) has grown significantly over the last few decades with EVs being widely regarded as a source of biomarkers for human health and disease with massive clinical potential. Secreted by every cell type in the body, EVs report on the internal cellular conditions across all tissue types. Their presence in readily accessible biofluids makes the potential of EV biosensing highly attractive as a noninvasive diagnostic platform via liquid biopsies. However, their small size (50–250 nm), inherent heterogeneity, and the complexity of the native biofluids introduce challenges for effective characterization, thus, limiting their clinical utility. This has led to a surge in the development of various novel EV biosensing techniques, with capabilities beyond those of conventional methods that have been directly transferred from cell biology. In this review, key detection principles used for EV biosensing are summarized, with a focus on some of the most recent and fundamental developments in the field over the last 5 years.

This article is categorized under:

Diagnostic Tools > Biosensing
Diagnostic Tools > In Vitro Nanoparticle‐Based Sensing

## Graphical abstract

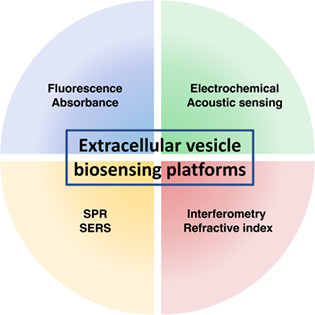

Various biosensing principles may serve for the detection and characterisation of extracellular vesicles. Herein, the key platform are systematically reviewed, with a focus on the most recent and fundamental developments.

## 1 INTRODUCTION

Every cell type in the human body secretes membrane‐bound nanoparticles, namely extracellular vesicles (EVs) (Doyle & Wang, 2019). Consequently, the size, shape, and molecular composition of EVs are incredibly varied, giving rise to unique subpopulations with a high degree of heterogeneity and complexity. The precise mechanisms involved in the packaging, biogenesis, and secretion of heterogeneous EVs by living cells remain widely unknown, but surface biomarker heterogeneity (transmembrane proteins, glycans, ligands, DNA, lipids) is likely a result of cell origin and functionality (Gudbergsson et al., 2019). The unique surface markers found in various EV populations may provide useful information towards disease detection and progression (Hilton & White, 2021) for a variety of diseases, including neurodegenerative disease (Bellingham et al., 2012), various cancer types (Belov et al., 2016; Worst et al., 2017), kidney disease (Thongboonkerd, 2020), and acute brain injury (Taylor & Gercel‐Taylor, 2014).

A ubiquitous abundance of heterogeneous EVs is found in all biofluids including plasma, serum, urine, saliva, breast milk, semen, and cerebrospinal fluid (CSF) (Maas et al., 2017), creating a plethora of EV‐rich sources. EVs have great clinical value in the context of monitoring overall patient health—distinguishing between healthy and diseased cells, determining the extent of disease progression, and assessing the efficacy of drug treatments (Hilton & White, 2021; Isola & Chen, 2017; Ullah et al., 2021). The potential for EVs to be disease biomarkers (Duijvesz et al., 2013), vehicles for drug or gene delivery, and as therapeutic agents for tissue regeneration make these supramolecular structures widely researched (Borges et al., 2013; Mathiyalagan & Sahoo, 2017). Identifying unique surface biomarkers present on diseased subpopulations of EVs will pave the way for novel and innovative tools to diagnose diseases and significantly limit their occurrence and mortality. Noninvasiveness and versatile collection, along with their phenotypic expression, has made EVs a coveted platform for liquid biopsies in diagnostics. With a view towards clinical applications, this review concisely summarizes recent and fundamental technology being used for EV characterization.

### 1.1 EV biogenesis and composition

**FIGURE 1:**
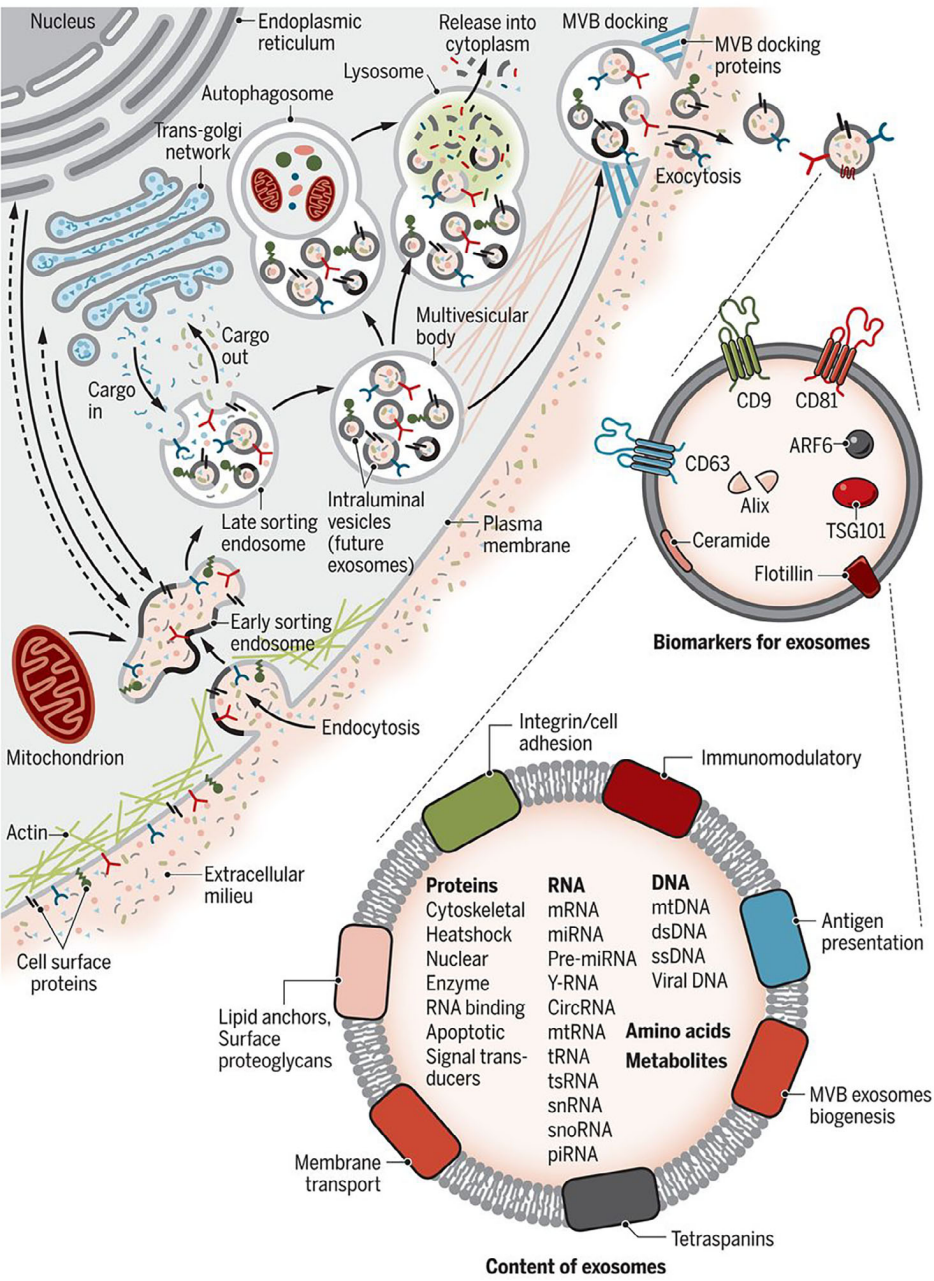
Schematic representation of extracellular vesicle biogenesis and biomolecular content. Reprinted with permission of Kalluri and LeBleu (2020)

The conserved mechanism of EV biogenesis in cells from all domains of life underpins their biological significance. EVs are formed via an exocytic pathway, involving the budding and fission of plasma membrane protrusions and can be endocytic in origin (Akers et al., 2016), resulting in a discrepancy in membranous and intravesicular content between EV subtypes. The endolysosomal pathway in a cell involves stimulus‐dependent formation of an early endosome, with eventual maturation into a late endosome (Hessvik & Llorente, 2018). Inward vaginations of clathrin‐coated microdomains form a multivesicle body (MVB), which is predisposed to one of three pathways: lysosome degradation, histocompatibility complex delivery, or fusion with the cell membrane resulting in the release of EVs (Figure 1) (Hessvik & Llorente, 2018). In most practical circumstances for diagnostic and therapeutic application, one cannot readily distinguish whether the isolated vesicles originated from the exocytic or endocytic pathways, thus the more general term EVs is commonly adopted. Moreover, when summarizing a study referring to “exosomes,” we default to the more generic term of EVs.

Advanced microscopy has allowed structural elucidation of EVs as spherical particles limited by a lipid bilayer membrane (van der Pol et al., 2014). This structure can change rapidly and dynamically according to underlying cellular processes, especially in response to cellular stress (Gudbergsson et al., 2019). EV functionality and role in disease is likely to result from their biomolecular composition, collectively known as EV cargo: a cytosolic mixture of proteins and nucleic acids, along with membrane integrated proteins, glycosaminoglycans and lipids. The database ExoCarta (http://www.exocarta.org) provides a cataloged repository of all reported proteins, lipids, and nucleic acids that have been identified in EVs (Simpson et al., 2012), which was added to the EV database Vesiclepedia (www.microvesicles.org/; Kalra et al., 2012). The repository underlines the influence of disease and cell type on EV content, exposing the significant heterogeneity in EV composition between populations (Ferguson & Nguyen, 2016). The inherent heterogeneity of EV subpopulations has been identified as a challenge that needs to be addressed in the field, and the technologies discussed here can allow for further elucidation of the enigmatic roles EVs play in human health and disease.

## 2 ATTRIBUTES OF EV BIOSENSING PLATFORMS

EV biosensing platforms must be sensitive enough to detect clinically relevant biomarker concentrations, often in the picomolar (pM) range. Additionally, high specificity is required to reduce the risk of false positive results and to discriminate sufficiently between disease‐state and healthy individuals. Other desirables include the minimization of diagnostic reagents, i.e. direct quantitative assessment of the analyte from minimal patient sample, thereby reducing the per assay cost. Most importantly, a key barrier to EV diagnostic development is the reproducibility of results between sites and assay runs, which is an essential consideration when attempting to establish a universal test method. Collectively, these challenges represent a significant clinical need, thus there is an expectation from clinicians for researchers to develop and apply novel biosensing techniques for biomarker validation, with the ultimate aim being to perform minimally invasive liquid biopsies for characterization of disease states using assays based on EV proteins.

These requirements can be delivered in the form of a biosensor, which are self‐contained analytical devices that measure biological or chemical reactions by generating signals that correlate to the presence of an analyte in a test sample. Typically, a biosensor is comprised of three components: (i) a bioreceptor that detects or interacts with a target analyte to generate a stimulus (biorecognition), (ii) a transducer that converts the stimulus into a signal, and (iii) a series of electronic components that processes, amplifies and conditions the signal for display (Mehrotra, 2016). In the following, we provide an overview of the most relevant studies for EV biosensing based on their detection method.

## 3 RECENT DEVELOPMENTS IN BIOSENSING METHODS FOR EV PROTEIN CHARACTERIZATION

### 3.1 Fluorescence

The measurement of fluorescence emission after the application of an excitation beam of light is termed fluorimetry and is an example of a spectroscopic technique. Fluorescence occurs when photons are absorbed by a molecule (fluorophore) from a ground electronic state to one of a few vibrational states within the excited electronic state of higher energy (Moldoveanu & David, 2013). Collisions with other molecules result in a loss in energy until the lowest vibrational state is reached, eventually dropping down to the ground state and resulting in photon emission at a particular frequency across the electromagnetic spectrum (Moldoveanu & David, 2013). Within a suitable concentration range, the intensity of emission is proportional to the quantity of the analyte‐bioreceptor conjugate, thus providing a method of quantifying the underlying marker of interest.

In the context of EV detection, J. Zhou et al. (2018) pioneered a fluorescence amplification procedure enabling detection of EV‐associated β‐amyloid‐(1‐42) oligomers. Aptamer–oligomer binding induced hybridization of replacement probes labeled with fluorescin amidite (FAM), and the subsequent oligomer release supported amplification cycles of the FAM fluorescent signal to give a limit of detection (LOD) of 20 pM. An aptamer‐based fluorescence assay was also reported by P. Zhang et al. (2019) with a LOD of 5 × 10^5^ particles/ml. The large mass of EVs was exploited to amplify the polarization of a fluorescent rotating species after associated aptamer ligands bound to EV surface antigens. Elsewhere, an aptamer‐linked H_2_O_2_ catalysis method was adopted by Dong et al. (2019) as part of the reported ExoID‐Chip. EVs exposed to biotinylated EV‐specific CD63‐aptamer probes were treated with streptavidin‐linked horseradish peroxidase (HRP). The addition of substrate 10‐acetyl‐3,7‐dihydroxy‐phenoxazine (ADHP) generated the highly fluorescent resorufin, displaying an outstanding LOD of 8.9 × 10^3^ EVs/ml in 10% (v/v) serum from breast cancer patients. FAM probe implementation was also reported by Kalimuthu et al. (2019) as part of fluorescence polarization detection of lipophilic dye 5‐dodecanoylamino fluorescein. The dye was inserted into the EV membrane, providing a single step assay with an LOD of 2.8 × 10^8^. The EV lipid membrane was also targeted by Dong et al. (2019) as part of a lateral flow assay. After modifying the EV membrane with biotin‐functionalized phosphatidylethanolamine (DSPE‐PEG‐Biotin), EVs were incubated with fluorescent nanospheres embedded with quantum dots. Nanosphere‐linked EV was flowed across a nitrocellulose strip producing a detectable fluorescent line upon capture, with a LOD of 2 × 10^6^ particles/ml in human saliva. Prior to these works, Xu et al. (2016) reported a simple method of EV membrane functionalization with CM‐Dil lipophilic dye. Labeled EVs were separated via gel filtration with eluent fractions monitored for fluorescence, displaying a LOD of 2.9 × 10^7^ EV sized particles/ml. Ibsen et al. (2017) combined lipophilic dyes with an alternating current electrokinetic (ACE) chip, to integrate EV isolation and detection of both surface and internal protein markers. EVs were enriched via their unique zeta‐potential, prior to labeling of the membrane CD63 and TSG101 markers. Lewis et al. (2018) applied this platform to detect glypican‐1 in EV from whole blood of pancreatic ductal adenocarcinoma patients. This study related fluorescence with EV concentrations and disease genotypes with 99% sensitivity and 85% specificity.

**FIGURE 2:**
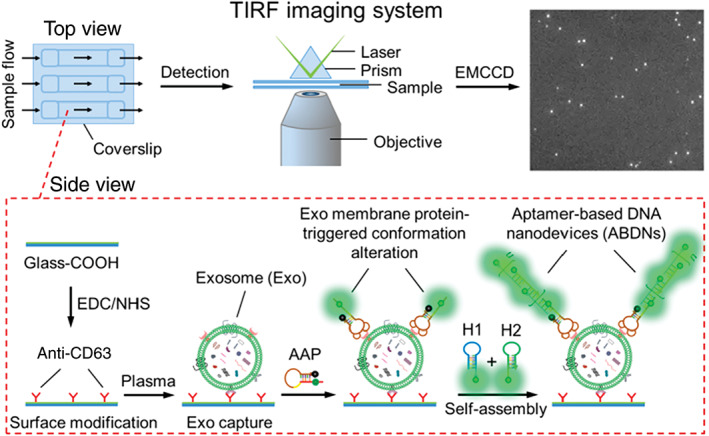
Schematic representation of ABDN‐TIRF assay for single extracellular vesicle visualization and detection. Reprinted with permission from He et al. (2019). Copyright 2019 American Chemical Society

While the aforementioned techniques focus on bulk EV analysis, single‐vesicle detection was demonstrated by K. Lee et al. (2018) via microfluidic multiplexed EV detection. EVs labeled with fluorescent antibodies specific to pan‐EV surface markers (CD9, CD63, and CD81) and gliobastoma tumor markers were imaged, using three fluorophores before being quenched with H_2_O_2_ and relabeled. This allowed identification of 14 distinct EV marker clusters at a single EV level. Single vesicle analysis was also achieved by He et al. (2019) using activable anti‐CD63 aptamer probes (Figure 2). Target protein tyrosine kinase‐7 (PTK7) was probed by the addition of a targeted aptamer probe. Reorganization upon aptamer binding to PTK7 caused fluorophore–quencher separation to produce a signal that was amplified by aptamer‐based DNA nanodevice self‐assembly. The signal was analyzed using total internal reflection fluorescence (TIRF) exhibiting a LOD of 1 × 10^6^ particles/ml (Figure 2). EV surface biomarker PTK‐7 was also targeted by J. Chen et al. (2020) as part of a label‐free aptasensor based on a hybridization displacement reaction. PTK‐7 recognition by aptamer sgc8 induced hybridization and displacement of a signal reporter, *N*‐methylmesoporphyrin IX, to emit high intensity fluorescence with a LOD of 3.4 × 10^8^ particles/ml in 30% (v/v) bovine serum.

**FIGURE 3:**
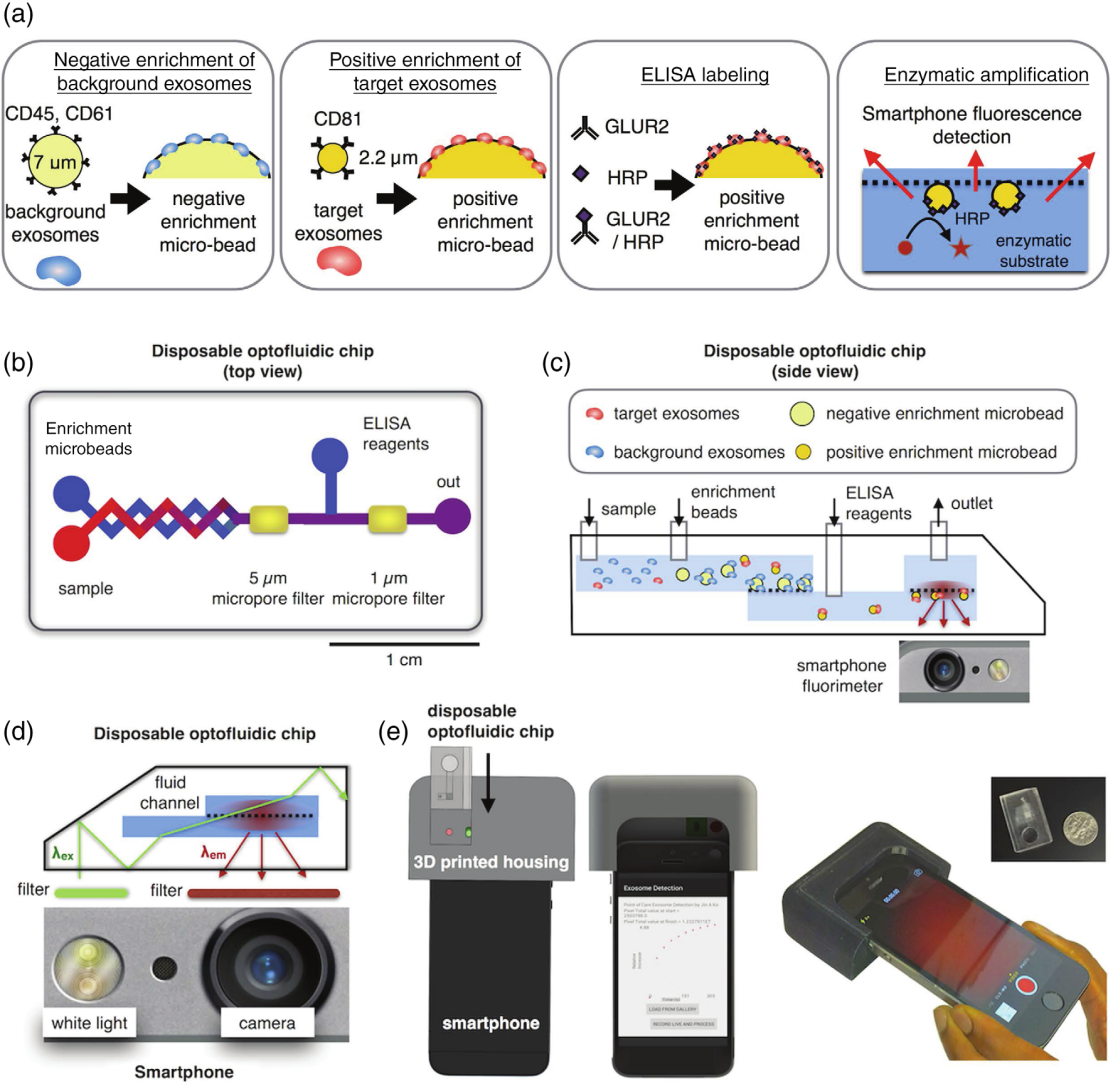
Design of a microbead‐based mobile extracellular vesicle (EV) detector. (a) Schematic overview of microbead immunoassay procedure from EV enrichment to fluorescent signal emission. (b) Graphical representation of optofluidic chip, and (c) a side view of chip and phone interplay with bead assay. (d) Set‐up of LED and smartphone camera for excitation and emission. (e) Rendered image of 3D‐mount for smartphone and disposable chip integration. Reproduced (adapted) under creative commons license from Ko et al. (2016)h

Tayebi et al. (2019) utilized molybdenum disulfide (MoS_2_)‐multiwall carbon nanotubes (MWCNTs) as part of a fluorescence resonance energy transfer (FRET) EV detection assay in order to exploit MWCNT fluorescence quenching properties of anti‐CD63‐PE (R‐phycoerythrin). Introduction of CD63‐positive EV overcame MWCNT‐PE binding to induce fluorescence recovery at a LOD of 1.5 × 10^6^ particles/ml. Y. S. Chen et al. (2019) combined membrane filtration and a magnetic‐bead based immunoassay to automate EV enrichment and quantification from human whole blood. Immunocaptured EVs underwent an on‐bead enzyme‐linked immunosorbent assay (ELISA), producing a fluorescent signal with a LOD of 3 × 10^10^ EVs/ml. A magnetic particle immunoassay based on fluorescence was also employed by H. Zhao et al. (2016) as part of the ExoSearch chip, offering multiplexed assessment of EV‐based tumor markers CA‐125, EpCAM and CD24, and the generic EV marker CD9. Effectiveness was displayed using just 20 μl of plasma and a LOD of 7.5 × 10^5^ particles/ml. Nonmagnetic photosensitizer beads were implemented by Yoshioka et al. (2014) as part of an amplified luminescent proximity assay, termed ExoScreen. Samples were incubated with acceptor beads and donor beads before being excited. Fluorescent emission occurred when beads were within a 200 nm proximity of each other, consistent with events of EV binding. W. Zhao et al. (2021) developed an ExoDEP chip where antibody functionalized beads captured EVs. The EV‐bound beads were then trapped in wells by electrodes due to their dielectric properties and fluorescently quantified. The reported LOD was 193 EVs/ml. Ko et al. (2016) used antibody conjugated microbeads to capture EVs prior to enzymatic labeling for fluorescence emission after excitation using a smartphone light emitting diode (LED) performing at a LOD of 10^7^ EVs/ml (Figure 3). The group integrated the optofluidic microchip and smartphone, thus taking advantage of “ready‐made” optics for application in point‐of‐care medical diagnostics.

A luminescence resonance energy transfer (LRET) assay was demonstrated by X. Chen et al. (2018) using rare‐earth doped upconversion nanoparticles (UCNPs) for their anti‐Stokes luminescent properties (ability to convert long‐wave excitation to short‐wave emission). Aptamer‐led recognition of EVs reduced the distance between a UCNP and a gold‐nanorod to increase LRET upconversion and luminescence quenching. This approach was able to detect EVs with a LOD of 1.1 × 10^6^ particles/ml. The same group adapted this technique to a “signal‐on” approach (Q. Wang et al., 2019). EV binding increased the proximity between tetramethyl rhodamine fluorophore and a UCNP, facilitating LRET to produce a fluorescent response, with a LOD of 8 × 10^4^ particles/ml. A proximity assay was also implemented by X. Zhao et al. (2020) as part of an aptamer‐cholesterol mediated ligation method (AcmPLA). EV‐aptamer conjugation enables cholesterol probe insertion into the bilayer membrane to initiate DNA ligation and FAM reporter hybridization. This approach was able to attain excellent LOD performance of 10^3^ particles/ml.

Xia et al. (2020) similarly inserted cholesterol‐linked DNA anchors with a HRP label into the bilayer membrane of captured CD63‐positive EVs. The addition of enzymatic substrates catalyzes the formation of 2,5‐di‐amino‐N,N‐bis‐(*p*‐aminophenyl)‐1,4‐benzoquinone di‐imine (PPDox), which quenches the fluorescence of fluorescein and initiates a color change from colorless to brown, thus also offering a colorimetric mode of EV quantitation. The LODs were 3.4 × 10^6^ and 3.1 × 10^6^ particles/ml for colorimetric and fluorescence measurements, respectively.

Thermophoresis‐assisted fluorescence detection is a technique that utilizes a laser‐induced temperature gradient coupled with diffusion and convection to isolate EVs by size within a microfluidic chamber (Huang, 2020; Li, 2021; Tian, 2021). Yang and coworkers combined tumor‐associated PD‐L1 aptamers with thermophoresis (HOLMES‐Exo_*PD*‐*L*1_) to quantitate circulating PD‐L1 levels to distinguish cancer patients from healthy patients with a 17.6 pg/ml LOD of EVs (Huang, 2020). Sun and coworkers demonstrated an aptamer‐based thermophoretic sensing (TAS) method with a variety of cancer‐associated proteins that has shown high sensitivity and specificity in detecting and discerning cancer types with an LOD of 10^7^ EVs/ml (Tian, 2021). The same group also recently developed a thermophoresis‐mediated DNA computation device which combined thermophoresis with fluorescent aptamer‐based logic gates for cancer‐associated protein markers, EpCAM and HER2 (Li, 2021; Tian, 2021). EVs were captured by CD63 aptamers bound on microbeads and accumulated by thermophoresis, diffusion, and convection. If both an EpCAM and HER2 aptamer bound to a captured EV, a toehold nanostructure is activated and formed between the two aptamers, triggering a hybrid chain reaction and resulting in amplified fluorescence. The resulting amplified fluorescence corresponded to targeted protein expression on captured EVs to discriminate between breast cancer and healthy patients with an LOD of 2.8 × 10^2^ EVs/ml.

The large number of successful examples of fluorescence‐based immunodetection is testament to the high signal to noise ratio, inexpensive single molecule sensitivity attributes, along with the scope for multiplexing and customizing arrays. Nonetheless, the requirement to have the analyte labeled with a fluorophore makes these assays susceptible to short lifespans, autofluorescence and issues with photostability, which has led to many researchers adopting absorbance‐based EV detection, as described below.

### 3.2 Absorbance

Absorption spectroscopy relies on the fraction of incident electromagnetic radiation that is absorbed by a material with a specific molecular composition. The measurement of photon absorption by a material at wavelengths within the ultraviolet (~190–380 nm), visible (~380–750 nm), or infrared (~750–2500 nm) regions, are termed UV absorption, colorimetry and infrared spectroscopy, respectively. Within a suitable concentration range, the chemical entity can be quantified through the Beer–Lambert law: *I*(*λ*) = *I*_0_(*λ*) ∙ exp[*α*(*λ*) ∙ *c* ∙ *L*], where, *I*_0_(*λ*) is the intensity of incident radiation, *α* denotes the absorption coefficient, *L* is the optical path length and *c* is the concentration of the sample (Swinehart, 1962).

Y. Zhou et al. (2020) implemented an aptamer‐based colorimetric assay to detect mucin‐1 positive EVs. By using a HRP‐mimicking DNAzyme sequence to elicit a color change upon EV binding, visual and quantitative measurements could be observed with a LOD of 3.9 × 10^5^ particles/ml. Elsewhere, aptamers have been combined with nanomaterials in order to enhance assay performance. Jiang et al. (2017) utilized an aptamer–gold nanoparticle complex that prevents aggregation in solution. The presence of EVs displaced the nanoparticle to initiate a color change from red to blue and characterized by visual assessment and absorption spectroscopy. Cholesterol‐linked aptamer probes were used by P. Zhang et al. (2019) as part of an amplified colorimetric assay, driven by alkaline phosphatase (ALP)‐induced silver ion reduction and deposition on gold‐nanorods. EV capture resulted in the metallization of gold that gave rise to a blue shift in the localized surface plasmon resonance, enabling visual and spectroscopic determination of EV concentration at LODs of 9 × 10^6^ particles/ml and 1.6 × 10^5^ particles/ml, respectively. Xia et al. (2017) also reported visible and colorimetric aptasening of EVs by combining CD63‐specific aptamers with single‐walled carbon nanotubes (s‐SWCNTs) that mimic peroxidase activity catalyzing H_2_O_2_ mediated oxidation of tetramethylbenzidine (TMB). EV binding displacing the aptamer from the s‐SWCNT induced a color change from deep blue via catalytic attenuation, which was quantified using absorbance spectroscopy with a LOD of 5.2 × 10^8^ particles/ml.

Y. Zhou et al. (2020) employed a custom chip in which EVs were captured on immobilized gold‐nanoparticles, prior to being exposed to detection antibodies and HRP for signal generation with a LOD of 9.5 × 10^4^ particles/ml. Moura et al. (2020) devised a magneto‐actuated immunoassay for EV detection from breast cancer cell lines. Bead bound EVs were tagged using anti‐CD24 and anti‐CD340 primary antibodies, followed by indirect and direct labeling with secondary antibodies and HRP‐linked anti‐CD63, respectively. This approach adequately overcame issues of matrix effects, receptor interference and performs at a LOD of 10^8^ EVs/ml in serum.

**FIGURE 4:**
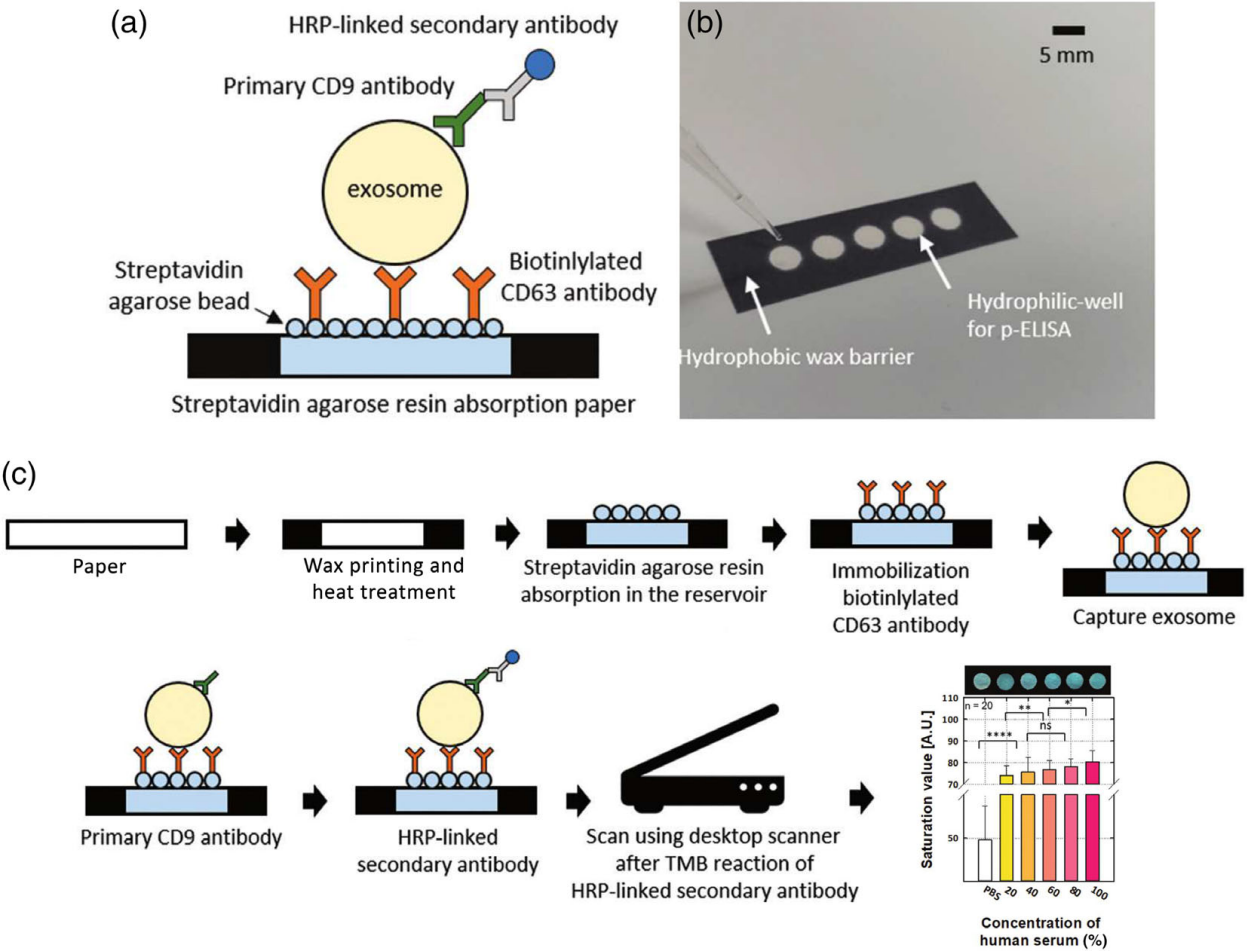
SABRI p‐ELISA principle. (a) Schematic representation and (b) optical image of p‐ELISA platform for EV detection. (c) Procedural steps from surface fabrication to result output. Reproduced (adapted) with permission of Royal Society of Chemistry from J. Lee et al. (2020)

Colorimetric‐based ELISAs have recently been translated to novel platforms such as paper‐based assays (J. Lee et al., 2020). Lee et al. devised a streptavidin agarose resin‐based immobilization (SABRI) approach to capture CD63‐positive EVs, prior to HRP conjugation and colorimetric read‐out (Figure 4). The appeal of a cost‐effective, paper‐based analytical device (PAD) has led to other research, notably from Chutvirasakul et al. (2020) exploiting the aggregation between immobilized EV‐capture vesicles (polydiacetylene [PDA]‐anti‐CD81) with CD81‐positive EVs. Solvent migration distance helped discern between samples of low‐ and high‐EV concentration, giving a LOD of 10^5^ particles/ml and a linear range of 10^6^–10^10^ particles/ml from only 1 μl sample volume and 6 min detection times. A PDA‐based liposome approach was also adopted by Kim and Lee (2019), by exploiting the blue to red colorimetric shift and fluorescence emission upon disturbance of the PDA structure, allowing measurement of EV concentrations at a LOD of 3 × 10^8^ vesicles/ml.

Like PADs, lateral flow immunoassays (LFIAs) have gained increasing prominence for EV detection due to their single step and cost‐effective qualities. LFIAs immobilize detection antibodies on a nitrocellulose membrane for analyte capture once flowed across the surface via capillary action. Oliveira‐Rodríguez et al. (2016) incubated EVs with anti‐CD63 conjugated gold nanoparticles before applying running buffer to induce sample migration, forming a visible line of bound gold nanoparticles that displayed a LOD of 8.5 × 10^8^ EVs/ml. More recently, the group applied LFIA for the detection of tumor‐antigens expressed in EVs, namely MHC class I chain‐related protein A (MICA) (López‐Cobo et al., 2018). The platform was able to detect poorly expressed EV‐sourced MICA in 25% (v/v) human serum at a concentration range of 5 × 10^10^ EV particles/μl.

In summation, EV detection principled on absorbance shares many of the advantages and drawbacks of fluorescence analysis. Favorable attributes include analyte quantification, flexible array sizes and specificities, inexpensive fabrication and relative simplicity of assay development. Some key drawbacks include measurements prone to interference from contamination and the solution conditions, which in‐turn affects the reproducibility of imaging.

### 3.3 Interferometry and refractive index

Other optical techniques for EV detection include interferometry and refractometry. Interferometry was implemented by Daaboul et al. (2016). for a multiplexed, label‐free single EV analysis. Shining visible light onto bound nanoparticles enabled assessment of interference in light reflection from the sensor surface, which is functionalized with antibodies against CD81 and CD63 tetraspanins, displaying a LOD of 5.1 × 10^9^ and 3.9 × 10^9^ particles/ml, respectively. Scope for clinical utility was demonstrated by successful detection from just 20 μl of human CSF. T. Wang et al. (2018) devised a microfluidic photonic crystal biosensor capable of detecting parasitic EVs based upon induced changes in refractive index. The crystal biosensor was fabricated with a subwavelength grating and a titanium oxide coating to achieve narrowband optical reflectance (reflecting a defined wavelength from a broad wavelength of incidence excitation). The capture of EVs caused a shift of the resonant reflection, with the net shift in the resonance wavelength linked to EV concentration at a LOD of 2.2 × 10^9^ EVs/ml. An advantage of these approaches is the avoidance of a label and the offering of a single‐step approach to detection. However, as seen in both cases, the achieved LOD is an order of magnitude above the 10^8^ EVs/ml commonly accepted upper limit of EV quantity in biofluids (Deville et al., 2021; Veerman et al., 2021), thus requiring amplification. A focus of the investigations reported herein is to ensure reliable detection of EVs at clinically relevant concentrations (1 × 10^8^ EVs/ml). An interferometric technique has since been commercialized by NanoView Biosciences and used to elucidate features of EVs at the single vesicle scale using protein or peptide capture (Gori et al., 2020; Mizenko et al., 2021).

### 3.4 Plasmon resonance

Another extensively researched platform for EV detection is based on surface plasmon resonance (SPR). SPR is a spectroscopic technique that detects analyte‐ligand interaction on a metal surface, most commonly gold. SPR studies real‐time changes in resonant oscillation of surface‐confined free electrons (referred to as surface plasmons) stimulated by plane polarized light passing through a glass prism at a specific incident angle (Homola et al., 2008). A key condition for surface plasmon excitement is the refractive index of the region proximal to the gold surface. Adsorption of biomolecular analytes induces changes in this interfacial refractive index, which results in alterations in plasmon excitement (Fang, 2017). This SPR response is defined as the shift in wavelength or angle of the “SPR minimum” in reflected light. When monitoring these temporal changes, binding kinetics can be derived, as well as the mass bound as a function of the surface area. SPR techniques have gained prominence due to their label‐free approach to determining mass‐uptake, compatibility with microfluidic set‐ups and a high sensitivity of measurement.

Rupert et al. (2014) initially demonstrated SPR detection of CD63‐positive EVs. EVs induced changes in resonance response as they bound to a capture antibody (thereby changing the refractive index of the adlayer). In addition, the sensing of a model liposomal system, coupled with a mathematical formalism enabled estimation of EV concentration. This work laid the foundation for dual wavelength excitation that enabled simultaneous measurement at two different sensing depths (Rupert et al., 2016). By using the ratio of these responses, film thickness, adsorbed mass and particle size were more precisely defined. Elsewhere, Sina et al. (2016) implemented SPR to detect human epidermal growth factor receptor 2 (HER2)‐positive EVs with a LOD of 2 × 10^6^ EVs/ml. The same group reported a more direct approach with no prior enrichment of EVs (Sina et al., 2019). The latter approach achieved a LOD of 8 × 10^6^ EVs/ml in more complex sample media in the form of undiluted serum.

**FIGURE 5:**
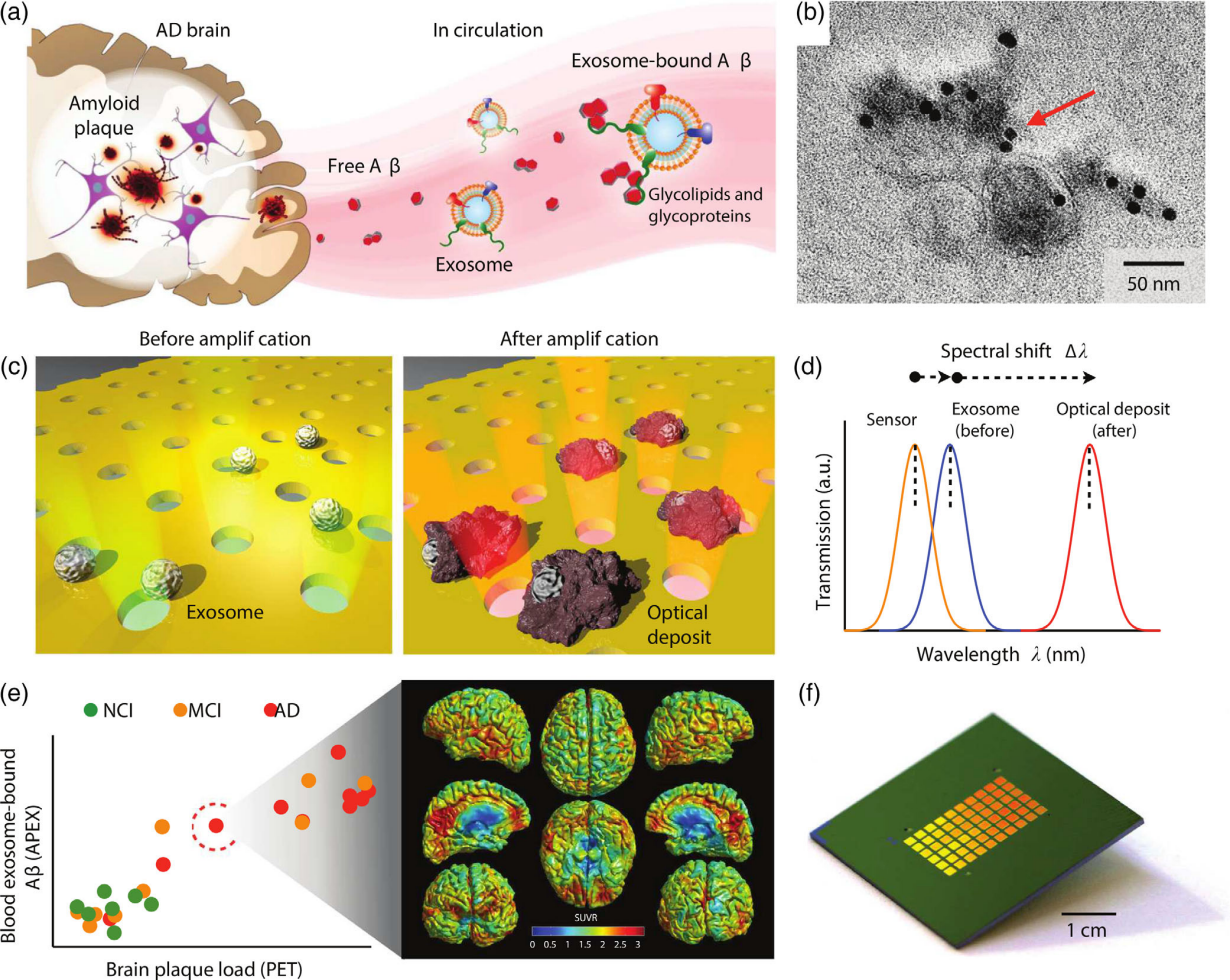
Summary of APEX platform for analysis of circulating extracellular vesicle (EV)‐bound amyloid‐β. (a) EVs associate with circulating amyloid‐β. (b) Confirmation of EV‐bound amyloid‐β using immunogold EM. (c) APEX assay schematic. EVs are immunocaptured onto a nanohole plasmonic surface. Using an in situ enzymatic amplification, insoluble optical deposits are locally formed on immobilized EVs. (d) Changes in the transmission spectra with APEX amplification. (e) APEX responses were correlated to PET imaging of brain amyloid plaque deposition. (f) A photograph of the APEX microarray. Reprinted under Creative Commons license from Lim et al. (2019)

SPR performance is reliant on the refractive index sensitivity, which can be tuned via nanostructuration on a sensor surface. Furthermore, SPR typically uses total internal reflection systems to monitor changes in the refractive index. In contrast, Im et al. reported a transmission‐based SPR assay using periodic nanoholes as sensitive yet discrete detection arrays (Kawecki & Ebert, 2004). The nanoplasmonic EV (nPLEX) sensor monitored the change in light transmission intensity and spectra upon EV binding at a LOD of 3 × 10^3^ EVs/ml. Instead of whole EV detection, Park et al. (2018) targeted internal proteins (AKT1) after EV lysis. Gold‐labeled proteins were immunocaptured offering signal amplification by interacting with the gold sensing surface via plasmonic coupling to display a LOD of 10^4^ particles/ml. Advancements of the nPLEX were published in 2019, termed the amplified plasmonic EV (APEX) (Figure 5) (Lim et al., 2019). APEX demonstrated that CD63‐positive EVs were able to sequester amyloid‐β directly from blood plasma. The change in transmission spectra and intensity was amplified by local deposition via an in situ enzymatic reaction, achieving a LOD of a mere 200 particles/ml. Alternatively, a 3D‐photonic crystal with a LOD of 1 × 10^4^ particles/ml was implemented by Zhu et al. (2018), using dual‐layered plasmonic nanostructures and optical cavity modes to enhance signal intensity via resonance coupling.

The incorporation of a camera as part of a SPR imaging (SPRi) based EV assay was reported by L. Zhu et al. (2014). The technique detected reflection changes at a fixed angle of incidence to ascertain differences in refractive index following EV binding to antibody microarrays specific to surface proteins (CD9, CD41b, MET). A similar method has been described by Picciolini et al. (2018) for the detection of generic EV markers (CD81, CD9) and neurological markers (CD171, ephrinB, PLP1, GM1). SPRi has since been advanced to the single EV level by Y. Yang et al. (2020). The technique, termed surface plasmon resonance microscopy, captured images with an inverted total internal reflection fluorescence microscope on the SPR biosensor. These images were subsequently processed using a deep learning algorithm for automated EV identification and quantitation, resolving concentrations as low as 1.8 × 10^8^ particles/ml. Raghu et al. (2018) published another example of single EV detection with a localized surface plasmon resonance platform using nanosized surface modifications. Using lithography, gold nanosensors were formed on the end of quartz pillars, performing as a microarray for multiplexed assessment. Integration with a camera enabled individual EV resolution at subfemtomolar EV concentrations.

Quantification of cancer‐derived EVs has also been described by Liang et al. (2017) via a nanoplasmon‐enhanced scattering (nPES) assay. Immobilized EVs were probed by gold nanospheres and nanorods, forming a gold sphere‐EV‐nanorod complex exploiting the influence of particle size and shape on light scattering. Reducing particle proximity to less than 200 nm (i.e., upon EV binding) induced coupled scattering of increased intensity, providing a sensitivity of 0.2 μg/ml. Liao et al. (2020) also used gold nanoparticles to amplify SPR signals in combination with DNA tetrahedron capture probes, displaying an LOD of 5.6 × 10^5^ particles/ml in 50% (v/v) human serum. Similarly, L. Wang et al. (2019) employed a double layer gold‐nanoparticle approach, whereby EV‐bound particles were hybridized with a secondary DNA‐linked gold‐nanoparticle to amplify the initial SPR signal in proportion to the bound EV concentration down to 5 × 10^3^ EVs/ml.

Plasmonic approaches generally rely on wavelength modulation, which demands costly and large spectrometers. Zeng et al. (2018) published an alternative technique using a plasmonic interferometer array sensor, using ring‐hole nanostructures for wavelength modulation, without the need for a spectrometer or an angle‐tuning prism. A LOD of 3.9 × 10^8^ EVs/ml was achieved using a desk‐top setup, compared to a LOD of 9.7 × 10^9^ EVs/ml using smartphone‐based detection. A similar approach was described by Y. Yang et al. (2018) for single EV analysis, termed interferometric plasmonic microscopy. Reflected and scattered light from a gold chip was collected, with the captured image being a product of the interference between the reflected light and the scattered surface plasmons. Each captured EV was distinguished as a bright spot captured on a frame‐by‐frame basis, helping to elucidate new mechanisms of immunointeraction such as the “hit‐stay‐run” phenomenon where EVs will interact with the antibody, briefly stay, and then dissociate. Liu et al. (2018) advanced the SPR set‐up in the form of an intensity modulated compact SPR sensor. The authors conceded a compromise in sensitivity (2 × 10^10^ particles/ml), but claimed superior quantitative performance compared to ELISAs.

Surface plasmon resonance can also be used to characterize EVs based on mechanical properties, such as rigidity. To this end, Caselli et al. (2021) developed a “plasmon‐based stiffness nanoruler” that measures membrane stiffness to uncover EV rigidity's influence on cell malignancy, given that rigidity of a vesicle correlates to its adhesion and uptake. When citrated gold nanoparticles (AuNPs) interact and adsorb to EV surfaces, the SPR profile of AuNPs changes, inducing aggregation that is proportional to EV stiffness. The extent of aggregation was then monitored by UV–Vis. This platform was validated on synthetic liposomes, which are well‐characterized, and subsequent testing on TRAMP‐C2‐derived EVs demonstrated a limit of detection around 10 nM.

In general, SPR exhibits very high sensitivity, often reporting some of the lowest LOD values in the field. In addition, it offers label‐free detection in real‐time and supports miniaturization and low cost fabrication. Due to the optical nature of the approach, SPR gives an indication of the dry mass of the bound adsorbate and is therefore not affected by coupled solvent. Furthermore, SPR analyses adsorption phenomena in a single mode of measurement, as opposed to the dual mode frequency or dissipation read‐out capabilities of competing technologies such as acoustic wave resonance with dissipation monitoring. A crucial consideration is the influence of colloidal contaminants in the sample and the disturbance they are known to induce in the refractive index at the sensing surface.

### 3.5 Surface enhanced Raman spectroscopy

Raman spectroscopy is a technique that relies on the vibrational modes of free molecules, providing a structural fingerprint of unknown entities. The principle is based on the inelastic scattering of photons at a Raman frequency ($\omega_{R}$) following irradiation of a vibrational system with incident light at a frequency of *ω*_0_ (Ding et al., 2016). The amplification of Raman signals using plasmonic coupled modes afforded by metal nanostructures is termed surface enhanced Raman spectroscopy (SERS), enabling single molecule detection. Here, metals with optical resonance properties are arranged to significantly enhance local electromagnetic fields as a result of excitation in the form of surface plasmon resonance (Ding et al., 2016). Enhancement in SERS occurs in two‐steps: (i) a local enhancement in electromagnetic field surrounding a plasmonic nanostructure or particle, transforming far field to near field at the *ω*_0_, and (ii) mutual excitation between the induced dipole of a molecule and nanoparticle gives rise to Raman polarizability derivatives that are up to three orders of magnitude larger than that of the free molecule (Smith & Rodger, 1999). In this case, the nanoparticles transfer the near field to the far field at the $\omega_{R}$.

In lower frequency vibrational modes of molecules, the incident, Raman scattering frequency and enhancement factors for (i) and (ii), (*G*_1_(*ω*_0_) and *G*_2_($\omega_{R}$)) are often comparable. Thus, the SERS enhancement factor is proportional to the enhancement of the local electrical field and incident electrical field (*E*_*loc*_ and *E*_0_ in the presence and absence of nanoparticles respectively) (Equation 1). The local electromagnetic field within interparticle gaps are intensified by strong electromagnetic coupling, with smaller gap sizes within the nanometer scale increasing SERS enhancement (Ding et al., 2016):

(1) $$G=G_{1}\left(\omega_{0}\right)G_{2}\left(\omega_{R}\right)=\frac{{\left(E_{\mathrm{loc}}\left(\omega_{0}\right)\right)}^{2}{\left(E_{\mathrm{loc}}\left(\omega_{R}\right)\right)}^{2}}{{\left(E_{0}\left(\omega_{0}\right)\right)}^{2}{\left(E_{0}\left(\omega_{R}\right)\right)}^{2}}\approx \frac{{\left(E_{\mathrm{loc}}\left(\omega_{R}\right)\right)}^{4}}{{\left(E_{0}\left(\omega_{0}\right)\right)}^{4}}$$

An early example of SERS for EV detection was reported by Tirinato et al. (2012) with the use of super‐hydrophobic surfaces functionalized atop arrays of silicon micro pillars. The hydrophobicity helped accumulate a large EV density within a small analytical footprint. The Raman fingerprint of proteins, lipids and nucleic acids allowed differentiation between healthy and colon cancer cell lines. Avella‐Oliver et al. (2017) integrated SERS with consumer compact disks, taking advantage of the silver coated nanosized grooves to reflect light for SERS monitoring. Silver nanoparticles bound to a thiolated peptide (LXY30) ligand were used by C. Lee et al. (2017) to detect α3β1 integrin‐positive EV. The EV‐LXY30‐SH‐silver nanoparticle complex generated a unique Raman fingerprint for EVs from different sources. Weng et al. (2018) reported an apta‐immunocomplex SERS assay using a gold coated magnetic bead and Raman reporters as a readout signal source. The presence of EVs reduced the SERS signal intensity from the reporter probe, performing at a LOD in the range of 3 × 10^4^–2 × 10^5^ EVs/ml.

**FIGURE 6:**
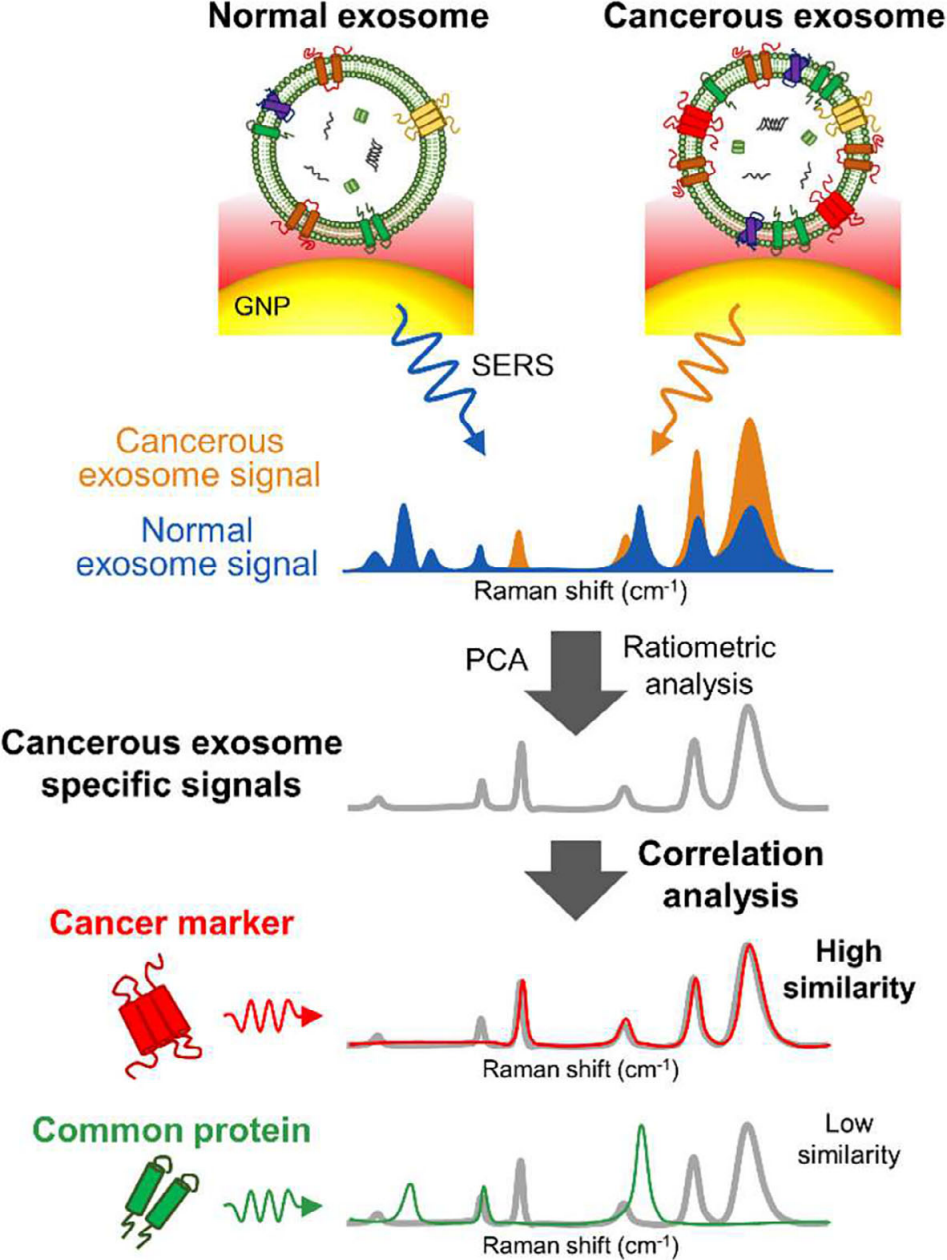
SERS‐based detection generates unique Raman scattering profiles that can be used to differentiate between cancerous and normal EVs. For comparison, EVs derived from lung cancer and normal cells were analyzed using AuNPs as plasmon‐active SERS signal amplifiers. Subsequent PCA served to perform correlation analysis on SERS spectra against profiles of known cancer marker proteins. Reprinted with permission from Shin et al. (2018). Copyright 2018 American Chemical Society

SERS detection of known disease biomarkers can be aided with principle component analysis (PCA) of Raman scattering profiles as described by Shin et al. (2018). Shin's study combined gold nanoparticle‐based SERS with statistical PCA to distinguish unique spectral features specific to EVs from cancer cells (EpCAM and EGFR), when compared to normal EVs and noncancerous proteins (Figure 6). EpCAM+ EVs were also detected by Kwizera et al. (2018) with a gold array device displaying a LOD of 2 × 10^6^ EVs/ml. The device comprised of small gold nanorods, where the anisotropic rod structure enhanced the electromagnetic field and SERS effect. The immunocaptured EVs were detected by the addition of a Raman reporter into a capture cetrimonium bromide (CTAB) capture layer. Raman reporters were developed by Tian et al. (2018) with an assay LOD of 2 × 10^4^ particles/ml. The group targeted EVs based upon their lipid bilayer and CD9 surface protein, via cholesterol‐assisted fixation of SERS nanoprobes and immuno‐capture processes respectively, creating a sandwich‐like complex to enhance Raman signals upon EV capture. M. Zhang et al. (2018) described the assembly of positively charged gold nanoparticles in hierarchical plasmonic structures using negatively charged triangular pyramid DNA (tetrahedrons). This created intense electromagnetic hot spots at the junctions between nanoparticles. The attachment of recognition probes assisted specific capture and quantification of EpCAM+ EVs down to a LOD of 1.1 × 10^5^ particles/ml.

Pang et al. (2020) reported a SERS immunoassay for the detection of EV‐based PD‐L1 using Fe_3_O_4_@TiO_2_ nanoparticles. The TiO_2_ shell bound to the hydrophilic phosphate head of the EV phospholipid membrane. The marker was subsequently probed using an anti‐PDL1 modified gold@silver@MBA SERS tag, with mercaptobenzoic acid (MBA) functioning as a signal reporter with a defined Raman peak. This method permitted phenotypic analysis from just 4 μl of sample and a LOD as low as 1 × 10^3^ particles/ml. The fast turnaround time of just 40‐min creates the prospect for this platform to be used in real‐time, tracking response to therapy and disease development. Rojalin et al. (2020) developed a highly sensitive SERS assay using a microscale biosilicate substrate with embedded silver nanoparticles to detect EVs. The biosilicate substrate acted to filter and trap EVs while the silver nanoparticles provided a drastic plasmonic enhancement to the inherently weak Raman signal. This substrate showed an incredible improvement in sensitivity with a limit of detection of near 600 EVs/ml, as well as quick acquisition times of around 1 s. Minimal concentrations EVs were required and rapid throughput of results makes this a promising biosensing platform for EV characterization. Koster et al. (2021) also reported the ability to electrostatically pull down EVs to commercial SERS substrates comprised of quartz microfibers decorated with gold nanoparticles, showing that the Raman signal could distinguish EVs from other contaminating co‐isoaltes, namely lipoprotein.

These reports all benefit from the limited sample preparation (label‐free) required for SERS‐based detection. Furthermore, the technique caters for rapid measurements with multiplexing scope and the possibility to be incorporated into portable devices. Additionally, the technique offers single molecule detection and fingerprint‐type analysis that enables complex molecular analysis. However, a fundamental limitation to SERS is the poor reproducibility between measurements and the extensive amount of substrate optimization required to counter this.

### 3.6 Electrochemical approaches

Alongside optical‐based detection, electrochemical‐based biosensors have become the method of choice for EV protein identification. Briefly, these platforms are based (in one form or another) on the relationship between a potential across a working electrode and electric currents generated from electron transfer via oxidative or reductive reactions of an electro‐active species/electrolyte. A common set‐up includes a potentiostat, a platinum counter electrode that serves to harmonize the total charge within an electrochemical set‐up, and a calomel or Ag/AgCl reference electrode, which receives a constant potential. Changes in electron transfer are induced upon analyte binding to a working electrode surface, which interferes with the flow of electrons across the electrode for current generation.

**FIGURE 7:**
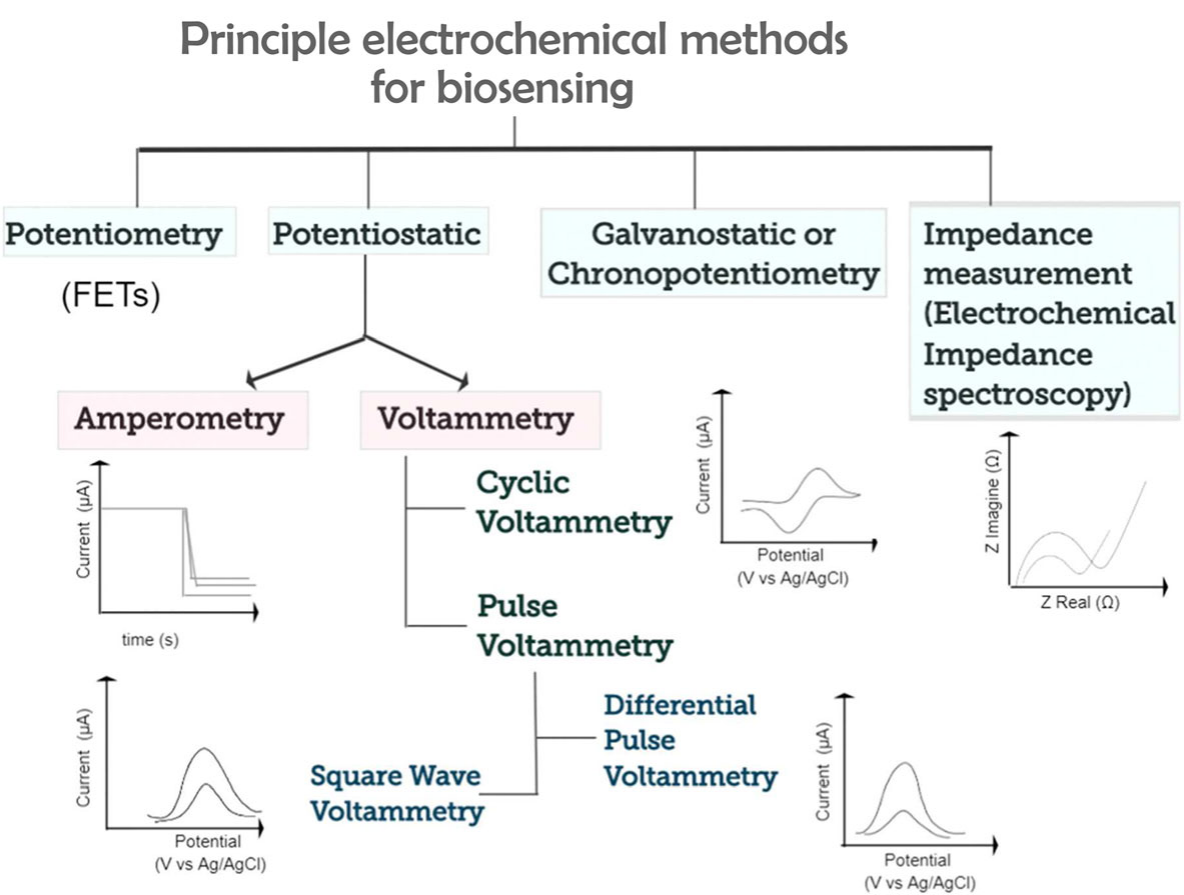
Graphical organization of commonly adopted electrochemical techniques used for biomarker detection, including E proteins. Adapted with permission from Vogiazi et al. (2019). Copyright 2019 American Chemical Society

These phenomena can be assessed in various modes of electrochemical measurement. Differential pulse voltammetry (DPV), electrochemical impedance spectroscopy (EIS) and amperometry are the three most commonly employed in the EV field (Figure 7) (Elgrishi et al., 2018). DPV studies redox properties via a series of voltage pulses across a linear potential sweep. The current difference at the working electrode before and after the pulse is measured as a function of potential. EIS measures the nonlinear response of impedance to perturbations in an electrochemical system. Impedance is a function of the increased resistance in working electrode polarization after analyte binding, which restricts charge transfer at the interface between the working electrode surface and electrolyte (Lasia, 2005). Amperometry relies on the generation and movement of ions in solution to measure the strength or changes in an induced electric current.

**FIGURE 8:**
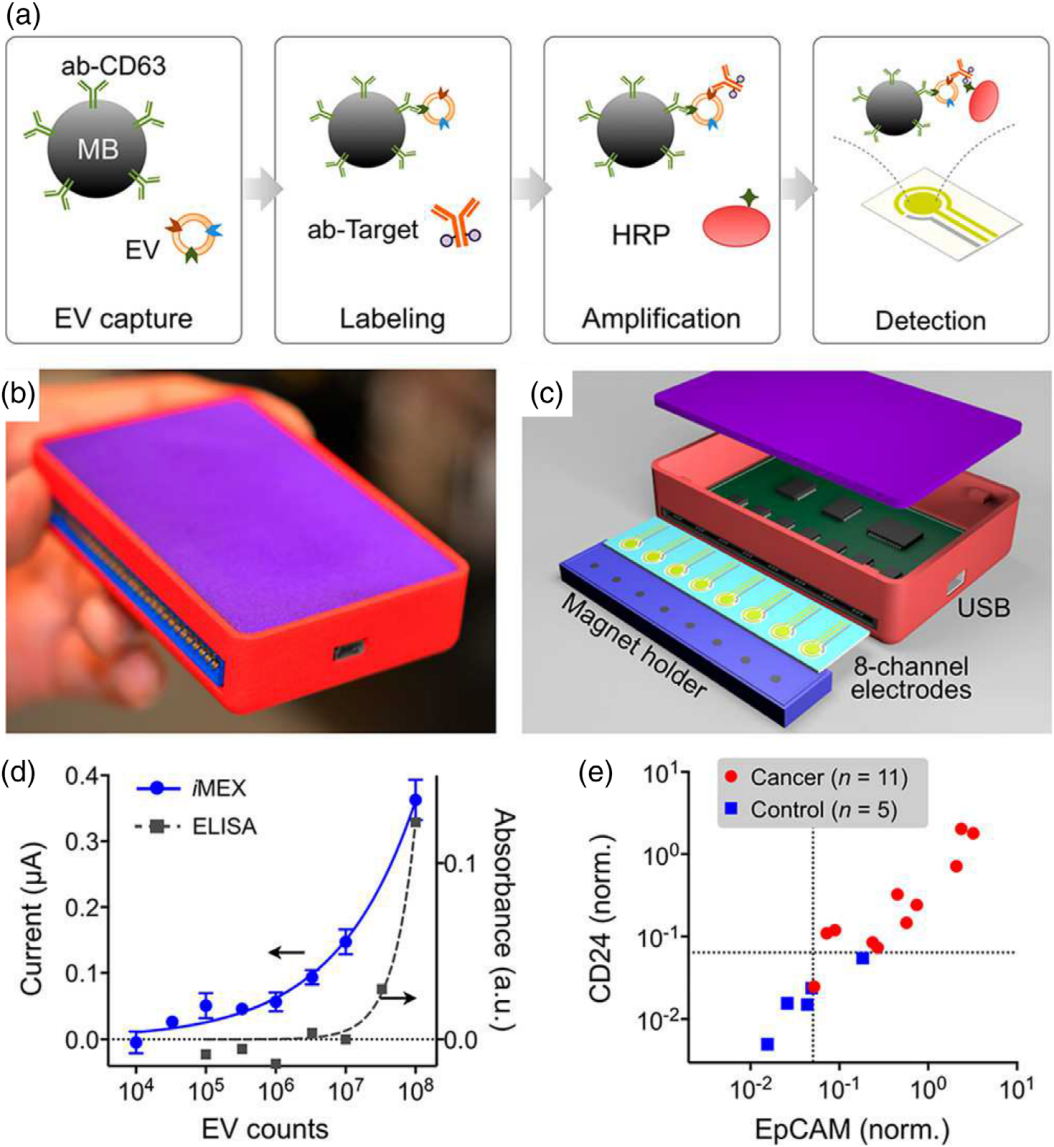
Summary of iMEX approach. (a) Schematic representation of iMEX assay. CD63‐ positive EVs are captured on magnetic beads in plasma and labeled with HRP for electrochemical detection. (b) The iMEX device. (c) Eight electrode set‐up of the iMEX. Magnets below the electrodes concentrate immunomagnetically captured EVs. (d) iMEX and ELISA response comparison to titrated concentrations of EVs. (e) iMEX analysis of plasma samples from ovarian cancer patients and healthy controls. Adapted with permission from Jeong et al. (2016) and Shao et al. (2018). Copyright 2016 and 2018 American Chemical Society

An early example of electrochemistry‐based EV detection was reported by Doldán et al. (2016) as part of a sandwich immunosensor approach. Immunocaptured EVs were labeled with HRP prior to TMB addition, which induced electrochemical reduction and passing of a current through a gold electrode. This fundamental study was able to detect EVs at a LOD of 2 × 10^5^ EVs/ml in up to 10% (v/v) serum concentration. A similar approach was employed by Jeong et al. (2016) in the form of an integrated assay device (iMEX) with eight‐channel electrodes for multiple marker profiling (Figure 8). The iMEX device performed with a LOD of 3 × 10^4^ particles/ml from just 10 μl of human plasma. Moura et al. (2020) reported an analogous approach for the detection of EV‐ markers CD9, CD63 and CD81, in addition to cancer‐specific EV markers (CD24, CD44, CD54, CD326). Hydroquinone was used as the electron mediator in place of TMB. As above, amperometry was used to measure the current generated, displaying an LOD of 10^8^ EVs/ml.

Aptamers have also found use as a capture ligand within electrochemical methods. Q. Zhou et al. (2016) published an early report on an electrochemical aptasensor for CD63‐positive EV detection, with a LOD of 10^6^ particles/ml. DPV was employed in combination with EIS by Kilic et al. (2018) as part of label‐free quantification of CD81‐positive EVs. A greater concentration of EV binding led to a greater barrier of electron transfer towards the working electrode, thereby reducing the oxidation peak and increasing the system resistance. Changes in EIS and DPV measurements were reported to have a LOD of just 77 and 379 EVs/ml respectively. EIS was adopted by Li et al. (2017) in order to detect both membrane located (CD81) and internal (syntenin) protein EV markers. Antibody functionalized gold beads were used as electrodes as part of impedimetric electroanalysis of intact vesicles (LOD of 1.9 × 10^5^ particles/ml) and for syntenin (post EV lysis) with a detection limit of 3–5 pM.

Cavallaro et al. (2019) described an electrokinetic method for label‐free EV detection. The procedure measures changes in streaming current upon EV binding within a microcapillary functionalized with antibodies specific to EV membrane proteins. The binding of an electrochemically active EV surface in the microfluidic channel dampens ion currents near the adsorbed layer. Quantifying the net change in the potential showed a dependence on particle size, surface coverage and concentration, sensitive down to 1.75 × 10^5^ particles/ml.

Elsewhere, the merging of amperometric and spectroscopic principles was demonstrated by Boriachek et al. (2019) using gold‐loaded nanoporous ferric oxide nanocubes, with an extremely low‐LOD of 10^3^ EVs/ml. The method exploits the superparamagnetic properties of the nanomaterial, which also possesses an intrinsic catalytic activity for the oxidation of chromogenic substrates such as TMB. Immunocapture of EVs generates a colorimetric and electrochemical signal in the presence of H_2_O_2_. Sun et al. (2020) reported a dual‐signal electrochemical approach for the detection of EVs derived from breast cancer cell lines. Black phosphorus nanosheets were formed with ferrocene‐doped metal–organic frameworks on indium tin oxide (ITO), creating a thin film. Methylene blue‐labeled CD63‐specific aptamers were linked with the ITO substrate to complete the detection platform. EV binding caused the aptamers to dissociate away from the ITO substrate, resulting in the methylene blue reducing its own oxidation–reduction potential, presenting a detection limit of 100 particles/ml. Reporting a similar LOD of 500 EVs/ml, W. Zhang et al. (2021) fabricated electrochemical microaptasensors that captured cancerous EpCAM+ EVs using CD63‐enriched microelectrodes and a microfluidic chamber. After capture, the EVs are then sandwiched by HRP complexes, creating a hybridization chain reaction amplification. Further amplification of the electrochemical signal comes from the current generated by the chemical reaction between the HRP complexes and the added 3,3′,5,5′‐tetramethylbenzidine (TMB)/H_2_O_2_. The generated electrochemical signal corresponded linearly to EV concentration.

The diverse range of electrochemical measurements are just one of the many desirable attributes for adoption of this principle as a biosensing technique. Their high sensitivity to analyte binding, simplicity of cell design, real‐time mode of analysis and scope for miniaturization lay grounds for translation into clinical use. Nonetheless, specificity is a limiting factor, with the surrounding bulk environment and ion content of the sample fluid influencing the result. Furthermore, the need for redox participants could negatively influence the biological integrity of ligand and analyte.

### 3.7 Electrochemiluminescence

Electrochemical reactions display clear utility in detecting EVs due to the redox and impedance signals one is able to measure. These electrochemical signals can be used to directly or indirectly generate a chemiluminescent output, a method known as electrochemiluminescent (ECL) biosensing. The ECL principle occurs via photon emission after the reaction of electrochemical intermediates. Redox reactions form reactive electrogenerated species on an electrode after the application of a sweeping potential. Excitation energy is obtained after the recombination of the oxidized and reduced species, resulting in the emission of light, which is proportional to the analyte concentration if the ECL‐active species was labeled to the analyte (Richter, 2008).

An ECL aptasensor was reported by Qiao et al. (2019) using mercaptopropionic acid (MPA)‐modified Eu^3+^ (luminescent probe)‐doped cadmium sulfide (CdS) nanocrystals as ECL emitters with H_2_O_2_ as a co‐reactant. Binding of CD63‐positive EVs resulted in the formation of a DNAzyme that catalyzed the reduction of H_2_O_2_, thereby reducing the ECL signal of the doped nanocrystal. ECL suppression displayed a LOD of 7.4 × 10^4^ particles/ml from human plasma. P. Zhang et al. (2019) employed Ti_3_C_2_ MXenes owing to their conductive and large surface area attributes, endowing them with electron transfer and catalysis properties. Electrodes functionalized with EpCAM‐specific aptamer captured EVs prior to Ti_3_C_2_ MXene detection. The addition of luminol as a reducing agent is able to generate a colorimetric signal without the need of traditional co‐reactors such as H_2_O_2_ and performed at a LOD of 1.3 × 10^5^ particles/ml. Recently, improved ECL efficiency has been demonstrated when the luminophore and co‐reactant are in close proximity, thereby shortening the electron transfer resistance and energy loss. Fang et al. (2020) employed black phosphorus quantum dots (BPQDs) for their optical and electronic properties to enhance the ECL intensity of $\mathrm{Ru}{\left(\mathrm{bpy}\right)}_{3}^{2+}$ (tris(bipyridine)ruthenium(II) chloride) by catalyzing its oxidation for photon emission. In addition, MXenes were used for support, probe immobilization and EV capture. With both MXenes and BPQDs possessing photothermal properties, a dual‐mode of measurement is offered at a LOD of 3.7 × 10^4^ EVs/ml. ECL as a detection principle exhibits high specificity or anti‐interference attributes, along with possessing a wide detection range. However, the process can suffer from electrode fouling, the requirement of numerous reagents, and multistep preparation.

### 3.8 Acoustic resonators

**FIGURE 9:**
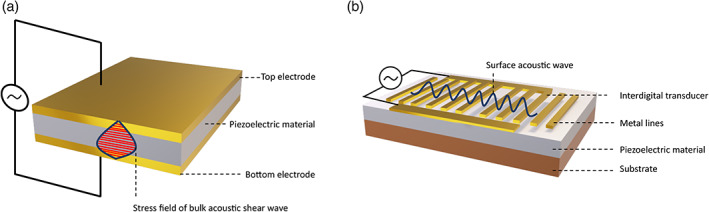
Schematic of a typical acoustic resonators. (a) Bulk acoustic wave (BAW) resonator, where acoustic waves propagate vertically through the entire 3D structure of the piezoelectric material following electrical field application. (b) Surface acoustic wave (SAW) resonator, composed of a comb‐shaped interdigital transducer (IDT) on a piezoelectric material. When an AC voltage is applied to the IDT, acoustic waves are generated, which are confined to the substrate surface (Rayleigh waves) and perpendicular to the IDT

An emerging analytical approach for EV characterization is based on acoustic resonance. These technologies are largely divided into those which utilize surface acoustic waves (SAWs) or bulk acoustic waves (BAW). Both involve the application of an alternating potential to a piezoelectric substrate, most commonly quartz. Piezoelectric‐based sensors are attractive for their ability to detect analytes directly in a sensitive, label‐free and real‐time manner. The most common BAW resonator used in liquids is a quartz crystal microbalance (QCM). QCM devices operate by generating thickness shear waves with frequencies between 1 and 10 MHz, where the entire piezoelectric substrate partakes in wave propagation (Rodahl & Kasemo, 1996). In contrast, SAW sensors oscillate via Rayleigh waves that are parallel to the surface, typically at higher frequencies (50 MHz to low GHz), with the energy being limited to the surface layer on the sensing substrate (Figure 9) (D'Amico & Verona, 1989). For both resonators, upon adsorbate binding to the oscillating substrate there is an induced change to the resonant frequency. Signal changes can be correlated to the physical characteristics and concentration of the analyte, as well as the physical properties of the bulk sample medium. When combined with a concurrent read‐out of dissipation (oscillator dampening), the mass, softness and viscoelasticity of the analyte can be determined via suitable models.

C. Wang et al. (2020) applied SAW principles to proteomic EV detection through the binding of surface‐CD63 proteins. Initial signals were further amplified by biotinylated‐antibody labeling of EpCAM antigen and the addition of streptavidin‐labeled gold‐nanoparticles, providing a LOD of 1.1 × 10^3^ particles/ml. Selectivity of the technique was demonstrated through the use of alternate capture antibodies, other vesicular bodies and nonspecific proteins, all exhibiting significantly lower responses when compared to the combination of anti‐CD63 and EVs alone. As a collective, these findings make acoustic‐based immunosensing a viable new technique as part of the EV characterization toolkit.

Stratton et al. (2014) published a quartz crystal microbalance with dissipation (QCM‐D) method for the detection of microvesicles (MVs) released from cells atop QCM sensors. While this approach provided new information on dynamics of exocytosis, it did not explore phenotypic EV detection via protein‐based characterization. Suthar et al. (2020) employed a BAW resonator to directly capture and characterize CD63‐positive EVs using a quartz crystal microbalance with dissipation monitoring (QCM‐D) without need for a secondary label. EVs at native concentrations were easily distinguished from co‐contaminants in complex media (75%, v/v; human serum) by selecting for EV mass, viscoelasticity, and surface antigens via specific changes in resonant frequency upon analyte adsorption. CD63‐positive EVs adsorb to the oscillating substrate, which resulted in a change in resonant frequency that correlated to the concentration and physical properties of CD63‐positive EVs. The change in resonant frequency upon CD63‐positive EV adsorption also correlated to physical properties of the surrounding bulk sample medium. The reported LOD was 1.4 × 10^8^ EV‐sized particles (ESPs)/ml for this technique, which allowed for direct phenotyping of EVs at native concentrations in complex media.

**FIGURE 10:**
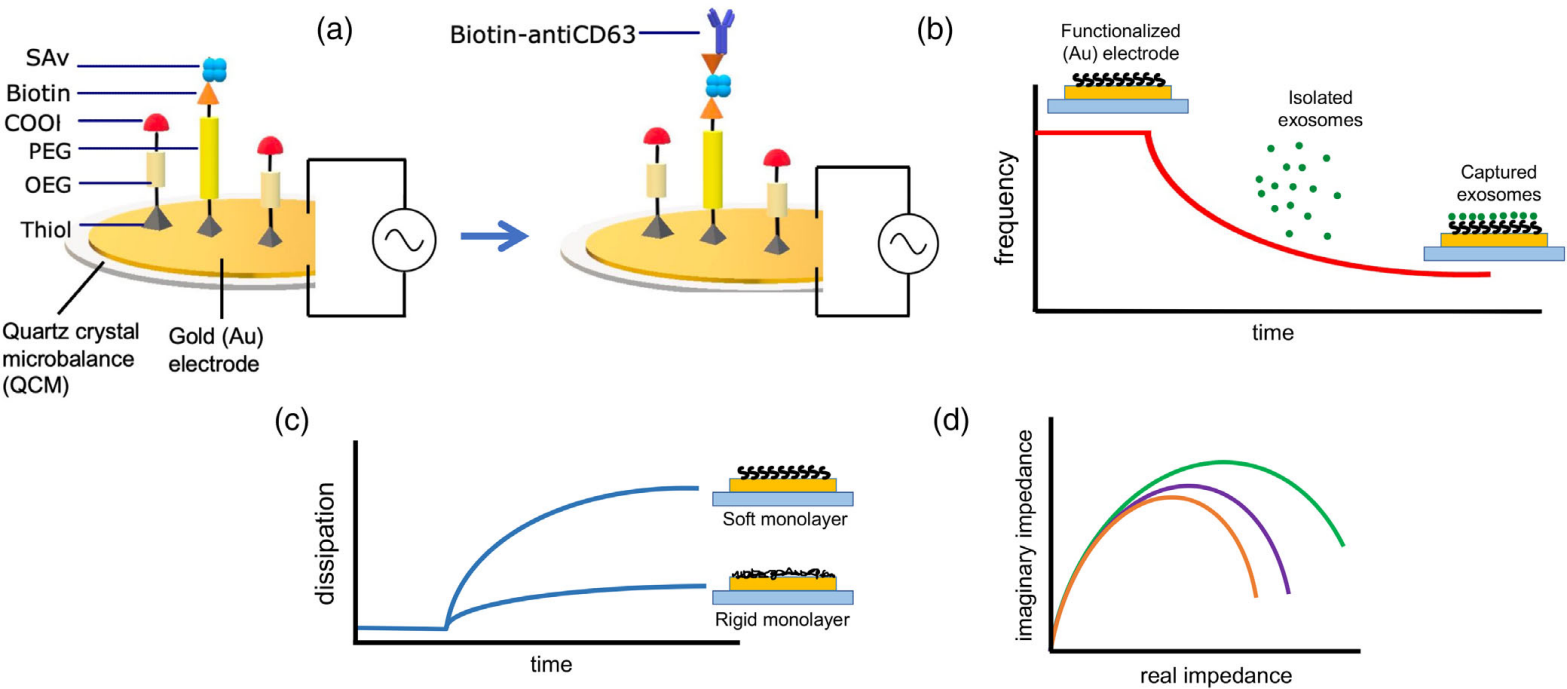
Electrochemical quartz crystal microbalance with dissipation monitoring (EQCM‐D) principle. (a) Schematic representation, (b) decrease in quartz oscillation frequency with increased EV adsorption, (c) voltage dissipation rate reflecting viscoelastic properties (soft and rigid) of adsorbed EVs, (d) EIS response of crystal quartz Au electrode (orange), functionalized crystal quartz Au electrode (purple), and EV adsorption (green)

Suthar, Prieto‐Simon et al. (2022) recently advanced QCM‐D based EV sensing by implementing the sensor as a working electrode within an electrochemical cell, collectively termed EQCM‐D. This instrumental setup enabled the incorporation of impedance spectroscopy, combining bulk acoustic wave techniques with electrochemical measurements to augment EV protein analysis, thus, using two distinct modes of characterization in tandem (Figure 10). A read‐out mechanism‐dependent LOD (ESPs/ml) in HEPES‐Buffered Saline (HBS) was obtained as follows: 1.7 × 10^8^ (QCM_*freq*_), 1.08 × 10^8^ (QCM_*diss*_), 5.34 × 10^7^ (EIS). Similar values were obtained complex biofluids (25%, v/v; serum) with 2.15 × 10^8^ (QCM_*freq*_), 1.25 × 10^8^ (QCM_*diss*_) and 6.71 × 10^7^ (EIS). QCM_*freq*_, QCM_*diss*_, and EIS refer to Figure 10b–d, respectively.

Although QCM‐D and EQCM‐D do not give the lowest LOD values in EV literature, the ability to acquire mass and viscoelastic properties of the bound analyte makes acoustic resonance an often synergistic technique for EV biosensor development. Moreover, QCM‐D platforms share many of the desirable criteria as set out by offering rapid, sensitive and affordable detection, in a label‐free and real‐time manner. QCM‐D signal generation is also well understood and extensively characterized across a breadth of applications. In spite of these characteristics and the studies mentioned, report on QCM‐D approaches for EV analysis remain scarce.

## 4 SUMMARY OF EV BIOSENSING TECHNIQUES

A summary of the major techniques to date on EV biosensing techniques for sample detection can be found in Table 1.

**TABLE 1:** Summary of biosensing techniques for bulk analysis of extracellular vesicles

Biosensing technique	Method	Advantages	Limitations	Reported limits of detection (EVs/ml)
Aptamer‐based colorimetric assay	Absorbance	• Visual and quantitative measurements • Enzyme‐free • Rapid throughput • Cost‐efficient • Sensitive	• Captures exosomes with specific antigens that are not ubiquitous to all exosomes	3.9 × 10^5^ (Zhou et al., 2020)
Colorimetric‐based ELISAs	Absorbance	• Visual and quantitative • Cost‐efficient • Sensitive • Rapid detection	• Sizes and types of EVs affect quantification (requires calibration)	10^5^ (Chutvirasakul et al., 2020); 3 × 10^8^ (Kim & Lee, 2019); 5 × 10^10^ (López‐Cobo et al., 2018)
Gold nanoparticles	Absorbance	• Visual and quantitative • Sensitive • Rapid detection	• Complex fabrication	9.5 × 10^4^ (Zhou et al., 2020); 1.6 × 10^5^ (Zhang et al., 2019); 8.5 × 10^5^ (Oliveira‐Rodríguez et al., 2016)
Magnetic bead‐based	Absorbance	• Direct assay in serum • Sensitive • Multiplexed detection • High throughput • Low maintenance	• Resource‐intensive instrumentation	10^8^ (Moura et al., 2020)
(MoS2)‐ multiwall carbon nanotubes	Absorbance	• Visual and quantitative measurements • Rapid detection	• Complex fabrication	5.2 × 10^8^ (Xia et al., 2017)
Acoustic resonators	Acoustic sensing	• Label‐free • Multiplexed detection • Real‐time • Specific and sensitive	• Limited selectivity • Antibody orientation may affect EV binding • Scarce EV‐related literature	1.1 × 10^3^ (C. Wang et al., 2020); 6.2 × 10^7^ (Suthar, Alvarez‐Fernandez, et al., 2022); 6.7 × 10^7^ (Suthar, Prieto‐Simon et al., 2022); 1.4 × 10^8^ (Suthar et al., 2020)
Electrochemic al assay	Electrochemical	• Rapid detection • High specificity • High sensitivity • Robust	• Purified EVs required as biologically relevant sample can create interference	77 (Kilic et al., 2018); 100 (Sun et al., 2020); 500 (Zhang et al., 2021); 10^3^ (Boriachek et al., 2019); 9 × 10^4^ (An et al., 2019); 1.8 × 10^5^ (Cavallaro et al., 2019); 1.9 × 10^5^ (Li et al., 2017); 10^8^ (Moura et al., 2020)
Electrochemilu mescence	Electrochemical	• Label‐free • Reproducible • Sensitive • Multiplexed detection	• Electrochemical setup • Requires optimization	3.7 × 10^4^ (Fang et al., 2020); 7.4 × 10^4^ (Qiao et al., 2019); 1.3 × 10^5^ (Zhang et al., 2019)
Antibody‐conjugated beads	Fluorescence	• Can use whole blood • No interference • Accurate identification and quantification	• Low capture rate • Resource‐intensive	193 (Zhao et al., 2021); 7.5 × 10^5^ (Zhao et al., 2016); 10^7^ (Ko et al., 2016); 3 × 10^10^ (Chen et al., 2019)
Luminescence resonance energy transfer	Fluorescence	• Portable • Disposable • Cost‐efficient • Sensitive	• Energy donor and receptor have specific conditions • Stability issues when EV binds to aptamer	8 × 10^4^ (L. Wang et al., 2019; Q. Wang et al., 2019); 1.1 × 10^6^ (Chen et al., 2018)
(MoS2)‐multiwall carbon nanotubes	Fluorescence	• High selectivity • Enzyme‐free • Rapid detection • Sensitive	• Complex fabrication	1.5 × 10^6^ (Tayebi et al., 2019)
Thermophores is‐assisted fluorescence detection	Fluorescence	• Low sample volume • Specific and sensitive • Rapid detection • Multiplexed detection • High throughput • Allows for simultaneous identification of EV proteins and RNAs	• Requires additional labeling for different protein markers of interest • Only EVs >30 nm are accumulated	10^7^ (Tian et al., 2021); 2.8 × 10^2^ (Li et al., 2021); 17.6 pg/ml (Huang et al., 2020)
Surface enhanced Raman spectroscopy	Plasmon resonance	• Label‐free • Real‐time • Highly sensitive • Multiplexed detection	• Resource‐intensive instrumentation • Low reproducibility	1 × 10^3^ (Pang et al., 2020); ∼600 (Rojalin et al., 2020); 2 × 10^4^ (Tian et al., 2018); 3 × 10^4^ (T. Wang et al., 2018); 1.1 × 10^5^ (Zhang et al., 2019); 2 × 10^6^ (Kwizera et al., 2018)
Surface plasmon resonance	Plasmon resonance	• Label‐free • Real‐time • Highly sensitive • Multiplexed detection	• Complex fabrication • Expensive instrumentation • Time‐consuming • Sensitive to pH, temperature, vibrations	3 × 10^3^ (Lim et al., 2019); 5 × 10^3^ L. Wang et al., 2019; Q. Wang et al., 2019); 10^4^ (Park et al., 2018); 5.6 × 10^5^ (Liao et al., 2020); 8 × 10^6^ (Sina et al., 2019); 1.8 × 10^8^ (Yang et al., 2020); 3.9 × 10^8^ (Zeng et al., 2018); 10 nM (Caselli et al., 2021)

An overview of techniques capable of individual EV particle analysis can be found in Table 2.

**TABLE 2:** Summary of biosensing techniques for single particle analysis of extracellular vesicles

Biosensing technique	EV characteristic	Advantages	Limitations	References
Atomic force microscopy	Size distribution, morphology, surface topography, and mechanical properties	• Minimal sample prep • High resolution • Measures EV stiffness and elasticity • Can be analyzed in solution	• Expensive equipment • Destructive • Low throughput • EVs must be immobilized to a surface • Nonquantitative	Beekman et al. (2019) and Sharma et al. (2020)
Cryogenic electron microscopy	Individual EV size and native morphology	• Minimal sample prep • High resolution • Accurate sizing/morpohology • Can use stains or labels to observe specific proteins	• Expensive equipment • Destructive • Low throughput • Nonquantitative	Emelyanov et al. (2020) and Cizmar and Yuana (2017)
Digital ELISA	Surface proteins	• Fluorescent labels can identify singular proteins of interest • Semi‐quantitative (estimate concentration)	• EVs must be isolated into single droplets for single EV analysis (can be done through microfluidics) • Expensive materials (i.e. antibodies) • No multiplexed detection for single EV	Liu et al. (2018) and Yang et al. (2022)
Digital PCR	Nucleic acid and surface proteins	• Quantitative • Multiplexed detection • Simultaneous tracking of multiple surface protein and RNA cargo	• EVs must be isolated into single droplets for single EV analysis (can be done through microfluidics) • Expensive equipment	Liu et al. (2021) and Ko et al. (2020)
Flow cytometry	Size distribution, surface/soluble markers	• High throughput • Low sample volume • Multiplexed detection	• Diffraction‐limited: >100 nm • Usually ideal for micron‐scale particles, rather than nanoparticles. Specialized flow cytometer is likely needed. • Cannot distinguish between EVs and aggregates • Typically requires fluorescent labeling	Campos‐Silva et al. (2019) and Görgens et al. (2019) and Tian et al. (2018)
Interferometric reflectance imaging sensing	Surface proteins and receptors	• Minimal sample prep • Nondestructive • High sensitivity • Low sample volume • Quantitative and qualitative • Multiplexed detection • High throughput	• Requires expensive analytical chips with conjugated antibodies • Size limitation: > 50 nm	Deng et al. (2022); Mizenko et al. (2021) and Yang et al. (2018)
Laser trapping Raman spectroscopy	Chemical fingerprint	• Minimal sample prep • Label‐free • Nondestructive • Low sample volume • Quantitative and qualitative • Direct imaging	• Long acquisition times • Low throughput • Expensive equipment	Carney et al. (2017), Enciso‐Martinez et al. (2020), and Penders et al. (2018, 2021)
Nanoparticle tracking analysis	Size distribution and concentration	• Minimal sample prep • Low sample concentration • Nondestructive • High throughput	• Performance fluenced by aggregates and larger nanoparticles • Cannot distinguish between EVs and protein aggregates • Size‐limited: >70 nm	Bachurski et al. (2019), Comfort et al. (2021), Maas et al. (2015) and Serrano‐Pertierra et al. (2020)
Resistive Pulse Sensing	Size distribution, concentration	• Quantitative • Does not rely on diffraction limited measurement • Analyzed in solution	• Size‐limited: >50 nm • Expensive equipment	Maas et al. (2017) and Vogel et al. (2017)
Scanning electron microscopy	Individual EV size, morphology, and surface topography	• High resolution • Direct imaging	• Expensive equipment • Destructive • Low throughput • Nonquantitative	Han et al. (2021) and Hartjes et al. (2019)
Surface enhanced Raman spectroscopy	Chemical fingerprint	• Minimal sample prep • Label‐free • Nondestructive • High sensitivity • Low sample volume • Rapid acquisition • High throughput	• Expensive lithographic substrates required • Issues with reproducibility	Jones et al. (2015) and Zhang et al. (2021)
Total internal reflection fluorescence microscopy	Surface/soluble markers, surface binding kinetics	• High throughput • Multiplexed detection • Quantitative • Direct imaging	• EVs must be immobilized at a surface • Expensive materials (i.e. antibodies)	He et al. (2019) and Zhou et al. (2020)
Transmission electron microscopy	Individual EV size, morphology, and inner structure	• High resolution • Direct imaging	• Expensive equipment • Extensive sample prep (staining, fixation) • Destructive • Low throughput • Expensive nanoparticle labels required for chemical specificity	Lennon et al. (2019) and Malenica et al. (2021)

## 5 OUTLOOK AND FUTURE PERSPECTIVES

As evidenced by biosensing platforms described in this review, there have been considerable improvements on the sensitivity and selectivity of EV detection. Nevertheless, current approaches mostly continue to require an expensive equipment set‐up, a complex fabrication process, or specific experimental conditions, which impair their clinical translation.

Though on the journey towards more simplified and translatable platforms, at this time, many biosensing platforms serve as invaluable research tools to detect EVs for further analysis rather than for clinical use. Over the past years, efforts were mainly drawn on analytical solutions to maximize selectivity, specificity and LOD of particular EV biomarkers. Future studies will need to focus on features of EV biosensing platforms that maximize clinical translation, i.e. lower cost modalities and nonexpert handling. The studies reviewed here typically are not validated in out‐of‐population studies, nor at multiple sites, thus, are firmly still proof‐of‐concept. To increase impact, future studies should examine significantly increased sample/patient numbers and emphasize on device reproducibility. Confounding factor of, for example, age, race, gender, BMI, are rarely considered in these early devices, limiting the potential for clinical adoption in the near term.

Given the high degree of heterogeneity among EVs, a growing need for single‐vesicle techniques to identify under‐represented subpopulations and possible bias in bulk analyses is evident. The techniques described in this review have largely been used for bulk EV analyses, as summarized in Table 1, and may greatly benefit from supplementary single‐vesicle analyses. Most EV studies utilize single‐vesicle techniques, such as nanoparticle tracking analysis and transmission electron microscopy, for basic EV characterization, including size distribution and concentration. In order to fully capture EV heterogeneity, a myriad of single‐vesicle techniques can be used which are briefly summarized in Table 2, many of which are specific variations of the bulk analysis techniques that have similar advantages but different limitations. These single‐vesicle techniques could improve specificity of disease diagnostics, though likely at the cost of sensitivity. Moreover, multiplexing is highly sought after as a result, which introduces a need for automated platforms with high‐throughput being able to process and analyze multiple EV preparations in under a few hours.

While EV biosensing platforms continue to improve and take in more data from higher number of samples/patients, capabilities of data analysis should scale accordingly. Capturing specific EV characteristics and the ability to associate different factors with aspects of health and disease will require methods for big data analysis. Therefore, machine learning is expected to be more routinely incorporated in EV characterization for robust interpretation.

Over the last few years, there has been a noticeable shift towards using plasmon resonance as a main detection principle of EV biosensing platforms. This may be because plasmonic platforms offer a nondestructive, label‐free, multiplexed, high‐throughput, cost‐effective, and incredibly sensitive method for both detecting and characterizing EVs. The main drawback of plasmonic resonance platforms is (as for many other current approaches) their requirement for expensive equipment. Realization of more affordable device architectures would bring EV biosensing platforms ever closer to clinical translation.

Variable detection sensitivities of EVs have been reported within each sensing strategy. Reasons for such variability may arise due to the lack of standardized EV isolation protocols, inconsistent definitions of EVs, different sources of EVs yielding varying degrees of heterogeneity, and different experimental parameters among studies using the same analytical technique. A significant hindrance to EV research is the lack of conclusive evidence to form standardized nomenclature or isolation protocols. For example, ultracentrifugation and size exclusion chromatography are considered gold standards among EV isolation techniques, however, these batch‐based techniques have been shown to yield a considerable amount of impurities and are lengthy to carry out. Compounded with inconsistencies in validation methods, this creates possible disparities between studies that may influence their utility. Moreover, these inconsistencies are illustrated by variations in reported EV sensitivities within each sensing strategy mentioned above. Nonstandardization of nomenclature and isolation protocols of highly heterogeneous EVs lead to inconsistent results that are not directly comparable between studies. This also highlights the utility in establishing standard calibration sets among EV‐sensing platforms to facilitate and validate reliable findings across multiple platforms. Having a set amount of specific EVs and commonly associated impurities mixed in a particular medium would represent a “standard” mixture of EVs for all instrumental calibrations and remove the effects of variability of EV heterogeneity, nomenclature, and isolation protocols on reported results. With more knowledge about EV properties and composition uncovered by existing and emerging biosensing platforms, we may finally standardize EV definitions and isolation protocols for reliable comparison and reproducibility between scientific findings.

As mentioned previously, isolation methods are a significant factor in the quality and accuracy of EV findings, because subpopulations may be excluded or diluted with biorelevant impurities. With a compromised sample population, the results may be biased or misleading. Several isolation methods are commonly used for the various biofluid sources, with different outcomes on purity, yield, specific EV surface proteins, etc. Such a diversity in isolation methods leads to findings that may not be widely applicable, and, thus, standardization of isolation methods will be important. A promising trend is also the development of integrated miniaturized platforms that combine EV isolation with characterization, e.g. using microfluidics. This would not only expedite diagnostic protocols but also greatly reduce the required sample volumes.

## 6 CONCLUSIONS

In this review, we have summarized recent applications of fundamental biosensing principles in the context of EV detection and characterization based upon their protein content. The various studies to date report promising results but come with some limitations. Often, the outcomes are not directly comparable to each other due to the lack of protocol standardization between studies. Furthermore, nomenclature and definition of EVs differ from study to study, since there is no official standardization in the field due to poor understanding of the heterogeneity of EV subpopulations. Additional convolution is caused by the various EV isolation methodologies that all have varying quality of EV yield and purity. Despite these caveats, the sensing platforms discussed above permit in many cases the selective detection and quantification of EVs at relevant physiological concentrations. In addition, new technologies are being developed to reduce complexity of usage and cost. With a growing portfolio in EV characterization methods, we also grow closer to being able to exploit EVs as biomarkers to diagnose and monitor the progression of disease.

## AUTHOR CONTRIBUTIONS

**Jugal Suthar:** Conceptualization (equal); methodology (lead); supervision (equal); writing – original draft (equal); writing – review and editing (equal). **Marissa Taub:** Formal analysis (lead); writing – review and editing (equal). **Randy Carney:** Writing – review and editing (equal). **Gareth Williams:** Conceptualization (equal); methodology (supporting); supervision (equal); writing – original draft (supporting); writing – review and editing (equal). **Stefan Guldin:** Conceptualization (equal); methodology (equal); supervision (equal); writing – original draft (supporting); writing – review and editing (equal).

## FUNDING

This work was funded by the Engineering and Physical Sciences Research Council (PhD studentship awarded to JS as part of the EPSRC Centre for Doctoral Training in Advanced Therapeutics & Nanomedicines, EP/L01646X/1, and to MT in the EPSRC and SFI Centre for Doctoral Training in Transformative Pharmaceutical Technologies, EP/S023054/1).

## CONFLICT OF INTEREST

Gareth Williams is an Associate Editor of the journal and was excluded from the peer‐review process and all editorial decisions related to the publication of this article.

## RELATED WIREs ARTICLES

Development and regulation of exosome‐based therapy products

Advances in imaging strategies for in vivo tracking of exosomes

## Data Availability

Data sharing is not applicable to this article as no new data were created or analyzed in this study
